# Allies not enemies—creating a more empathetic and uplifting patient experience through technology and art

**DOI:** 10.1007/s00066-024-02279-7

**Published:** 2024-09-11

**Authors:** Luca Tagliaferri, Bruno Fionda, Calogero Casà, Patrizia Cornacchione, Sara Scalise, Silvia Chiesa, Elisa Marconi, Loredana Dinapoli, Beatrice Di Capua, Daniela Pia Rosaria Chieffo, Fabio Marazzi, Vincenzo Frascino, Giuseppe Ferdinando Colloca, Vincenzo Valentini, Francesco Miccichè, Maria Antonietta Gambacorta

**Affiliations:** 1 UOC di Radioterapia Oncologica, Dipartimento di Diagnostica per Immagini, Radioterapia Oncologica ed Ematologia, Fondazione Policlinico Universitario A. Gemelli, IRCCS, Rome, Italy; 2 Dipartimento di Scienze Radiologiche ed Ematologiche, Università Cattolica del Sacro Cuore, Rome, Italy; 3 UOC di Radioterapia Oncologica, Ospedale Isola Tiberina—Gemelli Isola, Rome, Italy; 4 Università Cattolica del Sacro Cuore, Rome, Italy; 5 UOS di Psicologia Clinica, Fondazione Policlinico Universitario Agostino Gemelli IRCCS, Rome, Italy; 6 Centro di Eccellenza Oncologia Radioterapica e Medica e Radiologia, Ospedale Isola Tiberina—Gemelli Isola, Rome, Italy; 7 Scienze della Salute della Donna, del Bambino e di Sanità Pubblica, Università Cattolica del Sacro Cuore, Rome, Italy

**Keywords:** Quality of life, Digital health, Patient journey, Oncology, Care

## Abstract

**Objective:**

To understand whether art and technology (mainly conversational agents) may help oncology patients to experience a more humanized journey.

**Methods:**

This narrative review encompasses a comprehensive examination of the existing literature in this field by a multicenter, multidisciplinary, and multiprofessional team aiming to analyze the current developments and potential future directions of using art and technology for patient engagement.

**Results:**

We identified three major themes of patient engagement with art and three major themes of patient engagement with technologies. Two real-case scenarios are reported from our experience to practically envision how findings from the literature can be implemented in different contexts.

**Conclusion:**

Art therapy and technologies can be ancillary supports for healthcare professionals but are not substitutive of their expertise and responsibilities. Such tools may help to convey a more empathetic and uplifting patient journey if properly integrated within clinical practice, whereby the humanistic touch of medicine remains pivotal.

## Introduction

Cancer patients experience several challenges during their therapeutic journey, such as [1]

- *physical challenges*: pain, fatigue, stress, toxicity, hair loss, symptom management, etc.
- *psychosocial challenges*: existential earthquake, feeling anxiety and depression, questioning faith/hope, dealing with social stigma, changes in social relationships, loss of self-esteem, disruption of the personal (and family) life project, symptom understanding, etc.
- *organizational challenges*: care fragmentation, expenditures and financial toxicity [2], changes in lifestyle, etc.

While precision medicine focuses on complex phenotyping and multimodal target therapies (including the -omics) to reduce toxicity and side effects [3], personalized medicine aims at reintegrating all the aspects of the patient [4], including their psychological, social, and spiritual wellbeing. The main goal of personalized medicine is also participation, meaning that the patient should be empowered and enabled to make conscious choices about their health [5]. Therefore, it is important to open a multi-decision level, where patients can explore several dimensions of their health (physiological, psychological, social, spiritual, etc.). To achieve this goal, participatory medicine should follow the concept of value-based healthcare, in which the engagement of a patient is achieved when the clinician understands, engages, and gives back to the patient a re-elaborated story (and history) of care that must be validated within the professional relationship and through the experience of care.

Some tools can help the oncology team to address these challenges, such as art [6] and artificial intelligence (AI)-driven technologies [7, 8], and are intended as supports that provide an ancillary service to the standard of care while helping the patient to experience more uplifting and empathetic care.

This engaging experience can be subdivided into three phases:

- *Welcoming*: this is the phase when a patient enters the hospital. The team has the goal of enabling the patient to understand where they are as related to the context of their choice (the hospital, the clinician, etc.).
- *Cuddling*: the patient experiences a confirmation of their choice of being cured or not at that hospital (or dropout). The team must provide a participatory experience where the patient (and the family/caregivers) feel understood and empowered. It is fundamental to highlight that the healthcare professionals can create an experience where it is possible to give the relationship an “uplifting and empathetic” direction, but it must be accepted by and co-created with the patient (therapeutic alliance).
- *Reintegration*: this is the phase at which the patient can be reintegrated into normal life with new understandings of themself and the disease; such restitution is always co-created in the therapeutic relationship. Once in their new life, the patient should be enabled to testify to themself and to others the meaning of being cured and cared for.

With the endeavor of achieving humanistically guided medicine, where art and technology can be integrated into the standard of care without substituting the essence of the medical gestures, this narrative review aims at valorizing a value-based healthcare where the “care” dimension is a value co-created thanks to the participation of the person in their own therapeutic pathway, including the personalization of some parts of the therapeutic experience.

## Materials and methods

From October 2023 to December 2023, we conducted narrative review through a literature search in the PubMed database driven by the following research question: “How are art and technology used in oncology for patient engagement?” The search strategy is reported in Table 1.

**Table 1:** PubMed search strategy

**Database**	PubMed.ncbi.nlm.nih.gov
**Filters**	
*Publication date*	10 years
*Queries*	“art”[Title/Abstract] AND “oncology”[Title/Abstract] AND “patient”[Title/Abstract] “technology”[Title/Abstract] AND “oncology”[Title/Abstract] AND “patient”[Title/Abstract]
*Article type*	Clinical Trial
	Meta-Analysis
	Randomized Controlled Trial
	Review
	Systematic Review
*Article language*	English
**Results**	575 (136 for the first query on art, 439 for the second query on technology)

A multicenter research group belonging to the Fondazione Policlinico Universitario “A. Gemelli” IRCCS and to the Ospedale Isola Tiberina—Gemelli Isola was formed, designated to be multidisciplinary and multiprofessional by including radiation oncologists, onco-geriatricians, psycho-oncologists, pedagogists, and radiotherapy technicians. Within the group of radiation oncologists, physicians with experience in external beams, interventional radiation therapy (brachytherapy), treatment of pediatric patients, and digital health were included.

After finding 575 results (136 for art, 439 for technology), titles and abstracts were reviewed independently by the two centers, and duplicates were removed. A collective assessment was conducted in regular meetings, so that any disagreement between parties could be discussed and resolved within the research group. We finally selected a total of 100 articles (50 for art, 50 for technology) eligible for our review.

Results were thematically clustered into six major themes, three related to patient engagement through art and three related to patient engagement through digital technology. To contextualize a potential application in our centers, we further describe our experiences with integrating art and technology in the oncology patient’s journey (see “The Art4ART experience” section below).

Due to the nature of this review, a limitation is represented by the article selection process based on the subjective sensitivity of the group. Other articles not cited in this review may be valuable as well as those which have been chosen.

## The role of art-based oncology

The diagnosis of a noncommunicable disease such as cancer can be perceived as an existential earthquake, identified by Demetrio as a “watershed event” that marks a clear division between life before and after the discovery of the disease [9]. According to psychoanalyst Recalcati, when “dialoguing” with a piece of art, our unconscious can be attracted by something that “reason” is not always able to grasp, but this unexplainably (hence the mystery) constitutes an intrinsic part of how humans perceive the wonder and beauty of art [10, 11]. Considering art as an adjunctive therapy in the treatment of diseases [12], art therapy can be conceived as an *integrator of meaning*, a starting point to be shared with the clinician/therapist to symbolize the void of meaning left by a traumatic experience.

The World Health Organization (WHO) highlights several benefits of art therapy for oncology patients in terms of disease prevention, management, and treatment [13], especially physical and psychosocial. Such benefits are explored in the following paragraphs, in which we highlight three levels of patient engagement through art-in-oncology care.

### Appreciation

Appreciation means that patients do not perform any intentional action related to the artistic stimuli while “passively” enjoying it (except in terms of the physiological and functional activity of the body, e.g., mirror neurons).

Examples of art appreciation are exposure to famous paintings [14, 15] or listening to music records or poetry, stories, etc. This level of engagement can involve the integration of technology but is limited to providing art content (e.g., movies, videos, concerts) only used by the medical staff to this aim.

Appreciation of music/art and participation in events (concerts, famous paintings) have demonstrated physical benefits for cancer patients, such as alleviation of adverse side effects (e.g., drowsiness, loss of appetite, nausea) [16, 17], diminishing reliance on antiemetics [18], heart rate regulation [19], and fewer physical symptoms [20]. Such activities also contribute to reducing the psychological burden, such as by causing a reduction in anxiety and depression scores [15, 21], alleviating fatigue [22], reducing feelings of depression [23], heightening quality of life [24] and relaxation [25], and promoting new self-awareness [26]. The WHO suggests that these benefits may be enhanced by the role of arts in conveying pleasure, resilience, self-realization, and opportunities for learning and enhancing social relationships [13, 15, 19, 27–32].

At an organizational level, listening to music has also been documented to shorten the duration of hospital stay after cancer surgery [30], while art activities enhance communication with healthcare team and promote collaborative behaviors by patients [33–35], thereby enhancing social interactions and a sense of community [36]. Art engagement improves coping and psychological adaptation to the disease [16, 37, 38] and enhances optimism, spiritual satisfaction, and hope for survival [19, 21, 39] as well as fostering existential comfort and meaning and helping in saying goodbye [26].

In a deeper perspective, appreciation may require minimal interaction, meaning that the artistic stimuli can be chosen by the patient, and a more proactive engagement may be facilitated by technologies. For example, patients may choose to actively read poetry or books on certain topics or, in some cases, they can interact with technology (jukebox, tablets, monitors, smartphones, apps, etc.) that delivers the artistic stimuli to eventually select their preference.

Moreover, in a retrospective study that included glioblastoma patients, the role of spirituality and resilience in predicting clinical outcomes was evaluated by identifying, based on these parameters, two prognostic classes of patients in terms of 2‑year overall survival. Such results seem to suggest a possible effect of humanistic involvement on clinical outcomes (from attitude to emotional response) of the patient within their care pathway [40].

### Creation

Creation means that the patient is engaged in a form of enactment where the body is actively engaged in generating an artistic product. A practical example is artmaking, such as handcrafting (e.g., bricolage), playing music, creative writing, drawing, etc. Several studies highlight the benefits of regular dance and art-making activities, such as in terms of reducing pain [41, 42], especially with patients undergoing chemotherapy and stem cell transplantation [18].

It is important to consider that such activities require more than one session, structured in journeys conceived as “classes.” In particular, those which involve music, art (e.g., watercolor painting), poetry, and dance have been associated with positive therapeutic outcomes on patients’ mental health, such as lower levels of sadness, depression, and anxiety and higher levels of emotional wellbeing and quality of life [41, 43–51]. Interestingly, these outcomes are often related to physiological changes, including decreases in stress hormones (e.g., cortisol level) [52–54] blood pressure, heart rate, and inflammation [27, 44, 48]. Music therapy activities also induce a sense of community, improving relationships and communication with caregivers in palliative care [55, 56], while crafts and storytelling can support patients and their families with acquiring disease management strategies when reintegrated into their daily lives [57].

Another example of art creation by oncology patients is writing narratives (biographies, poetry). Sometimes facilitated by technologies like digital diaries, blogs, and platforms, this is an effective way to increase awareness among healthcare professionals about the disease condition, to enable them to provide further assistance or support to the patient when needed [58–60]. Creative activities also foster patients’ reflection on their cancer diagnosis [61] while facilitating the reconstruction of new biographical narratives [61].

Furthermore, art produced by patients can be a form of manifesto and witness to their illness experience that can be shared with other patients. For example, patient-produced photographs can give valuable insights to their supporting network, highlighting the need for connection, identity, and value in these existential earthquakes [62]. The “Pink Ambassador” initiative by the Italian Fondazione Umberto Veronesi is an example of patient advocacy promoted by former female patients who are devoted to developing culture, awareness, and education for preventing female cancers, often through art and sport initiatives [63].

### Immersive participation

Immersive participation is a form of engagement where the patient is absorbed in an experience involving multiple senses. These experiences can be conducted in groups or individually.

When provided collectively, immersion requires active participation by the patient, like dancing or singing. Participation is a form of immersion and creation. Dance can have rehabilitation purposes, but also a psychological value in terms of bodily perception [64], self-image, trust, identity, esteem, and consciousness [34, 41, 65, 66]. Similar outcomes have been noticed in choir sessions [33, 48, 49]. Physiological outcomes do not differ from what has been found in the previous literature (e.g., stress reduction, lower pain scores, regulated heart rate).

When provided in hospital settings, immersive stimuli like concerts, recorded music, or audio poetry can help reduce anxiety and depression among patients while promoting hope [67, 68]; it is worth mentioning that designing the setting aesthetically can make a difference also for workplace innovation [69], having an impact both on patients and healthcare professionals, such as the beauty of therapeutic gardens [1] and painted hospitals [6, 8].

Personalized immersive experiences can be enhanced through virtual/augmented reality (VR/AR) technologies, for example by projecting images of patients’ houses while they are receiving hospital-based palliative care to meet their desire to feel at home [70, 71]. Such technologies are used with patients receiving chemotherapy, confirming the reduction of anxiety and fatigue among patients [72, 73]. In the future, we could include the metaverse, but there is still little evidence on this topic.

## The role of AI-driven technologies in oncology

The contribution of AI-driven technologies (e.g., chatbots, deep learning platforms, apps) is rewriting the concept of assistance itself by retrieving real-world data through digital devices (phones, tablets, wearable devices, etc.) and modeling healthcare services in a more personalized way [74]. On the one hand, these tools can have organizational benefits for patients, such as improved care integration, and for healthcare professionals, including cost and time reduction [74, 75]. They also enable comprehensive process mining using real-world clinical data, which supports activities such as process discovery, conformance checking (e.g., adherence or non-adherence to a guideline), and process enhancement (bottleneck identification and solution) [76, 77]. On the other hand, a recent paper from the European Parliament highlighted several challenges that still need to be addressed [78]:

- Chatbots may alter the relationship between patients and clinicians.
- The relationship between patients and clinicians has issues of medical malpractice and negligence. The introduction of AI tools adds new complexity and actors in the patient–physician dynamic [79], which contributes to the complexity of the situation (e.g., decision-making processes).
- Physicians work according to clinical guidelines and technical standards. It is still unclear if the AI tools are systematically interoperable across clinical sites or whether they will be easily integrated into existing clinical workflows [80] and whether training programs are needed [81, 82].

In this scenario, we identified three levels of patient engagement with AI-driven technology in oncology: health remote monitoring, patient counselling and education, and patient management.

### Health remote monitoring

Like the *creativity* phase of art therapy, health remote monitoring is a level of engagement that requires the patient to wear a device (smartwatches, heart rate bands, digital rings, etc.) as a passive or low level of interaction. AI-driven technologies (e.g., wearable devices) can be used to retrieve patients’ biometrical and psychological parameters (e.g., blood pressure, sleep, heart rate, and patient-reported outcomes [PROMs] and measurements [PREMs]); such technologies are often connected with platforms (web-based or phone apps) useful for clinicians to detect otherwise unidentifiable symptoms (e.g., due to infrequency of visits, lack of communication in the intervals between visits, communication difficulties, and psychological aspects related to patients) early [83, 84], to better understand the patient’s health status and, eventually, to introduce preventative interventions if needed (e.g., schedule a hospital visit, lab tests, etc.) to avoid complications or worsening of symptoms.

With the pandemic, the integration of AI-driven technologies to support the pathway (*digital health*) of cancer patients played a pivotal role in delivering assistance and improving the patient experience and management [85–87]. Patient engagement can be increased as well, as they perceive being in control of their care. In addition, the system improved communication between patients and the healthcare team, creating a positive effect on the patient care delivery model [88]. This approach has resulted in better symptom control and improved physical function, demonstrating the potential of using technologies for improving patient care and possibly increasing survival outcomes for cancer patients during systemic therapies. The growing evidence on the usefulness of electronic patient monitoring using PROMs and PREMs justifies the increasing focus on these parameters in oncology drug efficacy evaluation studies [89].

### Patient counselling and education

Like the *creation* phase in art therapy, we envision patient counselling and education as a more proactive engagement to achieve a shift in meaning or behavior. Starting with classic websites, where content provision is almost exclusively one sided, i.e., from the institution to the patient, often in the absence of tools capable of offering facilitated or personalized navigation based on the patient’s clinical condition or pathology [90], the innovative technologies that offer interaction with conversational agents (e.g., chatbots) allow a new form of empowerment because patients can interact with technologies to receive preliminary support and are enabled to make a change in their perspective or behavior.

The Rosa chatbot was created to provide first genetic counselling information to patients at risk of hereditary breast and ovarian cancer. Quality-assured information was available 24/7, and the communication of this digital companion was tailored to be reassuring; however, all participants confirmed that the figure of the genetic counsellor cannot be replaced if hereditary cancer is confirmed [91].

In a recent study in patients with gastrointestinal cancer, researchers reported that patients texted back to the chatbot after postinterventional follow-up at least once to seek additional information by texting the words “chemo” or “support,” discovering that fatigue and neuropathy were the most frequently reported symptoms and finding that patients were satisfied with the chatbot, which was perceived as being user friendly and able to give valuable information [92].

Chatbots are mainly designed to mimic human-like communication with users seeking answers to questions about their symptoms and treatment and other useful information [93]. Conversational agents can give information that is perceived to be of comparable value to that offered by physicians, having the potential to assist people with minor health concerns and thereby optimizing time resources by mitigating the necessity for medical consultation. Consequently, this may give physicians more time to spend with patients who need their consultation the most [94]. Such tools can also be trained to guide patients with breast cancer requesting a consultation with psycho-oncologist as well as to improve the medication adherence rate [95].

Chatbots in oncology care seem to demonstrate high user satisfaction compared to the standard of care, improving patient-centered communication through accessible cancer-related information; hence, research in this field needs extensive testing and improvement before being implemented in clinical practice [96]. Automated chats can help patients with head and neck cancer to understand and self-manage their symptoms [97].

Delivery systems like chatbots could be created to have an “empathetic” personality, and their communication could be more personified as a digital health strategy to improve user satisfaction, engagement, and dialog quality [98]. Great attention is given to design algorithms and linguistic models that provide tailored communication while conveying a mirroring speech that emulates empathy [99], often with the goal of building trust and helping the patient become more confident with the tool to improve the therapeutic alliance and personalize treatments.

A large body of literature is focused on patient mental health education, not only in oncology [100–102]. Breast cancer patients can improve self-care behaviors and reduce chemotherapy side effects through a chatbot that provides personalized education and real-time accessibility to high-quality information, which has been useful for nurses in educating patients taking an active role in managing their symptoms [103]; moreover, quality of life of breast cancer patients can be improved using audiovisual intervention to enhance patient education [104]. Other uses of chatbots are being developed in healthcare for educating patients in achieving behavioral changes [105] such as healthy lifestyle behaviors [106, 107], and it is possible to co-create a “healthbot” with patients to help them adhere to quitting smoking [108].

### Patient management

Like the *immersive participation* phase in art therapy, some technologies can be fully integrated within the daily routine of the patient as a form of integrated care. Home-based rehabilitation after a chronic disease is often difficult to manage due to a lack of continuity in care between the hospital and homecare. Strategies to prevent and reduce the risk of dropout and lack of adherence are essential to optimize the quality of life of patients [109].

This comprises the field of digital therapeutics (DTx) that aims at reducing the costs of care while providing a boost in healthcare by using real-world data to unravel patients’ composite lifestyle biomarkers [110, 111]. Such technologies can empower patients for secondary prevention or postinterventional follow-up. Oncology patients may fail to adhere to the post-discharge clinical pathway if informational, clinical, and emotional support is lacking for their reintegration into their new lifestyle. This period could last for months, and it is often interrupted by the transition from home to hospital and vice versa [109].

Research into DTx still requires consolidated evidence, but some aids like virtual coaches could help patients manage and adapt their life in adherence to the care plan. For example, the “digital excipient” can be virtual assistants, reminders, reward systems, etc., and they are designed to allow patients’ adherence to the post-discharge clinical pathway [75]. The integration of a digital diary into the oncology patient’s journey may help clinicians access PROMs and PREMs related to the patient’s mental health status, perception of care, disease awareness, and self-empowerment, thereby enhancing communication and the overall relationship between patients and providers [58, 83].

## The Art4ART experience

Guided by the belief that art can provide uplifting experiences to cancer patients and that supervised technologies can enhance the patient journey by providing ancillary care, we report our experience named “Art4ART.”

This experience was originally built upon the scientific, educational, and clinical legacy of the Radiation Therapy Unit at the Fondazione Policlinico Universitario “A. Gemelli” IRCCS [6] with the name of “Gemelli ART” and the goal of alleviating what oncology patients experience as an existential earthquake. Specifically, we believe that the deepest and most unspoken aspirations of patients (physical, mental, social, and spiritual) must be welcomed, cared for, and guided by healthcare professionals toward a holistic approach that empowers patients to adopt a new lifestyle.

In our vision, art serves as a vehicle to facilitate the process of sensemaking of the disease and its challenges and acts as an activator of empowerment in patients, thus promoting their physical, social, psychological, and spiritual wellbeing. Technology is a tool that aims to meet these needs (sometimes by providing artistic stimuli; sometimes by retrieving PROMs and PREMs) as an ancillary resource of the clinicians to ameliorate the outcomes from the standard of care. In this context, reporting a concrete example, a humanization of care protocol has been developed for patients who are to undergo interventional radiation therapy (brachytherapy) for gynecologic cancer to identify patients’ needs and promote psychological wellbeing (the HAPPY protocol). The results identified eight interventions to be implemented, including having access to more information before procedures and the need for explanations using simpler words, e.g., “interventional room” should be preferred to “bunker” [136]. Anxiety and fear could be decreased through psychological support and the creation of a comfortable environment, also by the installation of a monitor in the treatment room on which the patient could listen to music or watch videos. The extension to a second phase of the study validated the effectiveness of the HAPPY protocol and its important role in patient management by observing an improvement in anxiety/depression; the obtained results are in line with data in the literature [137].

The following paragraphs will briefly describe the experiences with pediatric patients, with geriatric patients, and with those who need psychological assistance at the Gemelli ART Department (Fondazione Policlinico Universitario A. Gemelli IRCCS). Paragraph “The Isola ART experience and the Terentium project” will introduce the example of an ancillary project starting with the Radiation Oncology Unit of the Ospedale Isola Tiberina—Gemelli Isola, in Rome.

### Pediatric patients

Regarding pediatric oncology, either alone or combined with surgery or chemotherapy, RT plays a pivotal role in treating various malignancies [112, 113]. The evolution of technology and recent scientific breakthroughs has not only facilitated the reduction of early and late side effects [114] but also propelled the development of standardized multicenter experimental protocols.

However, pediatric patients undergoing RT face additional challenges. Although not physically painful, close collaboration with pediatric patients is essential, first during the preparatory phase (immobilization, position maintenance, and seclusion), and then during the treatment delivery phase, as these phases often elicit distress reactions [115, 116]. Stress reactions, pain, anxiety, and depression are common among children with cancer, posing a formidable challenge for healthcare providers. This underscores the necessity of a dedicated and personalized workflow for pediatric patients [117, 118].

Additionally, when patients struggle to maintain a fixed and reproducible position, sedation or general anesthesia (GA) becomes a requirement [119]. This introduces several challenges, including lifestyle changes for the family (e.g., restrictions on food and drink) and an elevated risk of medical complications. Minimizing the use of sedation may not only benefit healthcare costs but also enhance the overall experience for patients and their families. Psychological interventions have demonstrated efficacy in terms of preventing negative reactions in children and reducing the reliance on sedation during radiotherapy [120–122].

**Fig. 1:**
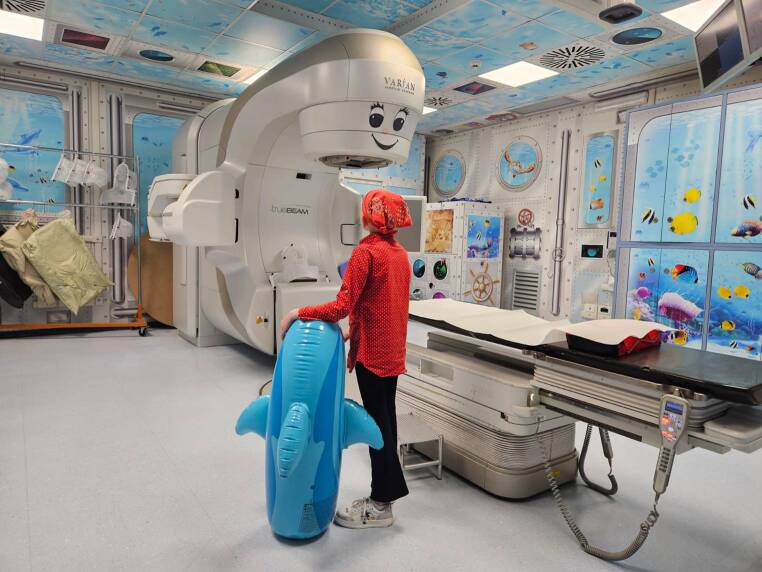
Image of the setting

At the Gemelli ART Department, the RADAR project was initiated to enhance the personalization of pediatric RT through a biopsychosocial care approach. Drawing inspiration from a marine setting artistically depicted on the treatment room walls (Fig. 1), the project employs assessment tools such as the Multidimensional Assessment for Pediatric Patients in Radiotherapy (MAP-RT) schedule, age-appropriate psychological preparation, psychological support, creative activities, and video tools [123].

**Fig. 2:**
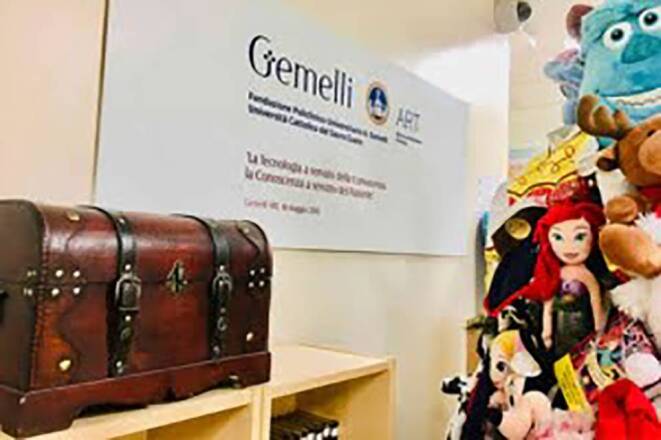
Image of the Dreams Chest

These interventions strive to place the patient and family at the center by fostering engagement and co-creation processes in RT. Among RADAR’s activities, one of the interventions most appreciated by patients and parents is grounded in the principles of the token economy and the reward system in RT, aptly named “The Dreams Chest” (Fig. 2). It provides pediatric patients the chance to choose a present online to be received on the last day of their therapy.

Daily RT sessions serve as “tokens” towards reaching this treasure, with the ultimate goal representing the child’s dream. This personalized and online-chosen object, within a fixed budget, holds significant personal value. In recent years, over 400 children have expressed their dreams through this project, thanks to generous donors from both large and small companies. The Dreams Chest has emerged as an economically sustainable method that contributes to increased adherence to RT in pediatric patients.

Since the inception of the RADAR project in 2018, the use of anesthesia procedures has seen a significant reduction, translating into lower healthcare costs. The sedation rate dropped from 19% to 13%, resulting in an average annual saving of 45,000 euros [8]. In our ongoing efforts to monitor childcare, we have incorporated the Parents PedSQL^TM^ Healthcare Satisfaction Hematology/Oncology Module. This tool (after Italian translation and cultural adaptation) demonstrated a high level of general satisfaction as well as high levels of satisfaction in the domains of information, inclusion of family, communication, technical skills, and emotional needs [124].

Additionally, other clinical monitoring programs involving technicians, psychologists, and radiation therapists are employed to capture the health needs of pediatric patients and their parents. Looking forward, we plan to introduce a social robot (SR) capable of staying inside the room with patients in 2024, thereby supporting young patients in engagement and compliance and influencing emotional wellbeing.

### Aging population

Regarding elderly patients, it is known that aging of the general population and the impact of the so-called “silver tsunami” on cancer care requires the creation of dedicated care pathways that guarantee sustainability of care. According to a recent estimate, 14 million new cancer diagnoses are expected in people over the age of 65 years by 2035, accounting for 60% of the global cancer incidence [125].

Defining a care pathway for the elderly person must include a comprehensive assessment that considers the comorbidities, functional reserve, life expectancy, and social and economic background of that person. Comprehensive geriatric assessment provides stratification of patient’s risk of toxicity and identifies those patients who need individualized care and intensive supportive care (vulnerable and frail patients) [126, 127].

In geriatric oncology, the challenge is to treat vulnerable and frail patients, i.e., those patients with lower functional reserve, with the aim of providing a tailor-made treatment strategy, which, through appropriate supportive care, ensures the best therapeutic result with the least impact on physical and cognitive performance and quality of life.

Personalized therapies include not only the customization of doses and treatment schedules but also the context of care in terms of places of care, clinicians involved, and services that can assist the healthcare provider in the clinical pathway.

Among these facilities, telemedicine is unevenly employed in geriatric settings to monitor vital parameters, symptoms, and performance of elderly patients. Its ability to intervene widely where healthcare resources do not allow for the constant presence of a doctor or nurse has permitted transfer of the assistance of many elderly patients to the comfort of their home and enabled the gaps in assistance between hospital visits to be filled. Early evidence in oncology suggests the usefulness of telemonitoring in the elderly oncology patient [128].

At the Gemelli ART Department, elderly patients are the focus of the clinical pathway called “Cristallo” (Crystal). This dedicated pathway sets up patient management within a multidisciplinary team [129] with access to the center’s technologies, treatment locations enriched with natural and artistic elements, and the artistic content on a digital platform specifically designed for our hospital [6].

Furthermore, a ward dedicated to treatments and supportive care for frail patients has been created. The ward includes an immersive room where, through visual, auditory, and sensory stimuli, it is possible to reduce the level of distress perceived by patients at various stages of the treatment pathway.

The immersive room is also designed to support patients with mild to moderate cognitive impairment during treatments, where hospital and cancer treatments can lead to a deterioration in cognitive status. Through stimulation of the patient with familiar environments and sounds, maintenance of cognitive function and reduction of behavioral disorders are promoted.

### Conventional and art-guided psycho-oncological support at Gemelli ART

When starting cancer treatments, numerous patients often experience heightened levels of pain and emotional distress as well as a decline in their quality of life (QoL), regardless of age. Psychosocial distress tends to be consistently elevated both during and after radiation therapy (RT) [130]. A recommended approach to improve psychological wellbeing involves connecting patients with diverse coping mechanisms through psychosocial support programs. The importance of integration of nonconventional medical treatments such as art therapy alongside standard approaches is emerging in the scientific scenario [6, 8]. Among the forms of art that are attracting attention in oncological research, cinema and music stand out.

As anticipated in our review, there is evidence regarding *distraction therapy* with relaxing and engaging content, such as concerts, fascinating places, exotic beaches, for managing cancer treatment side effects and providing specific psychological benefits [131]. Additionally, watching movies tailored to specific oncological populations allows the emergence of positive and negative emotions, their sharing in a group, and facilitates the recognition and expression of experiences and feelings [132].

Another form of art explored in RT is music. Data regarding the effect of music, particularly on anxiety, are currently limited and controversial. Some studies show an effect on anxiety [133], while others do not [134]. In general, the scientific community is unanimous in thinking that listening to music might provide comfort and distraction in the RT context [135]. Music therapy might best address the significant levels of anxiety and distress in patients undergoing RT, along with the RT session disruption, patient movement during treatment, and the need for repositioning they might engender [135].

**Fig. 3:**
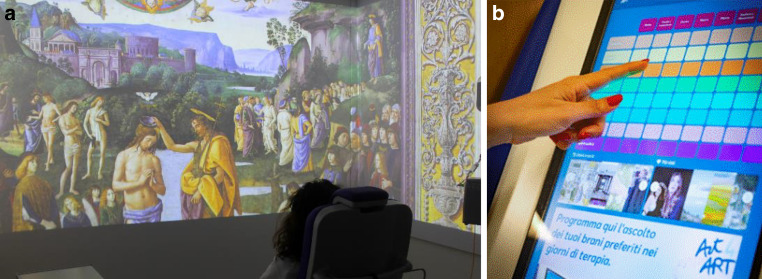
**a** Immersive chemotherapy infusion room;** b** “juke box” application for choosing artistic entertainment content

At Gemelli ART, we plan to implement cinema and music in a personalized manner, allowing the patient to be the protagonist by choosing their preferred form of art and its content. On the other hand, watching a movie during chemotherapy could alleviate the associated symptoms (Fig. 3). Applied to widely represented conditions in the Gemelli ART Department, such as breast or gynecological cancer, this approach could provide us with data on approximately 1000 patients per year. Being able to choose what to listen to and which movie to watch can be an empowering resource, and this, ultimately, can improve the resilience and psychological profile of these patients.

At Gemelli ART, psychological support and the use of art as a means to improve patients’ wellbeing are managed by psycho-oncologists. The psycho-oncologists follow patients across the entire RT course and carry out specific psychological questionnaires. Psychological support is personalized for each patient, with a minimum of three sessions (typical schedule) and a maximum of eight sessions (intensive schedule), depending on each psychological profile, clinical evaluation, and individual requests.

Personalized psychological support consists of three standalone phases: i) processing emotions related to the cancer diagnosis and recovering psychological wellbeing by improving the patient’s resilience, ii) addressing RT with emotional and cognitive coping strategies to manage side effects and psychological effects, and iii) organizing personal strategies at the end of RT treatment to support restarting everyday life and/or beginning a new therapeutic approach [138].

**Fig. 4:**
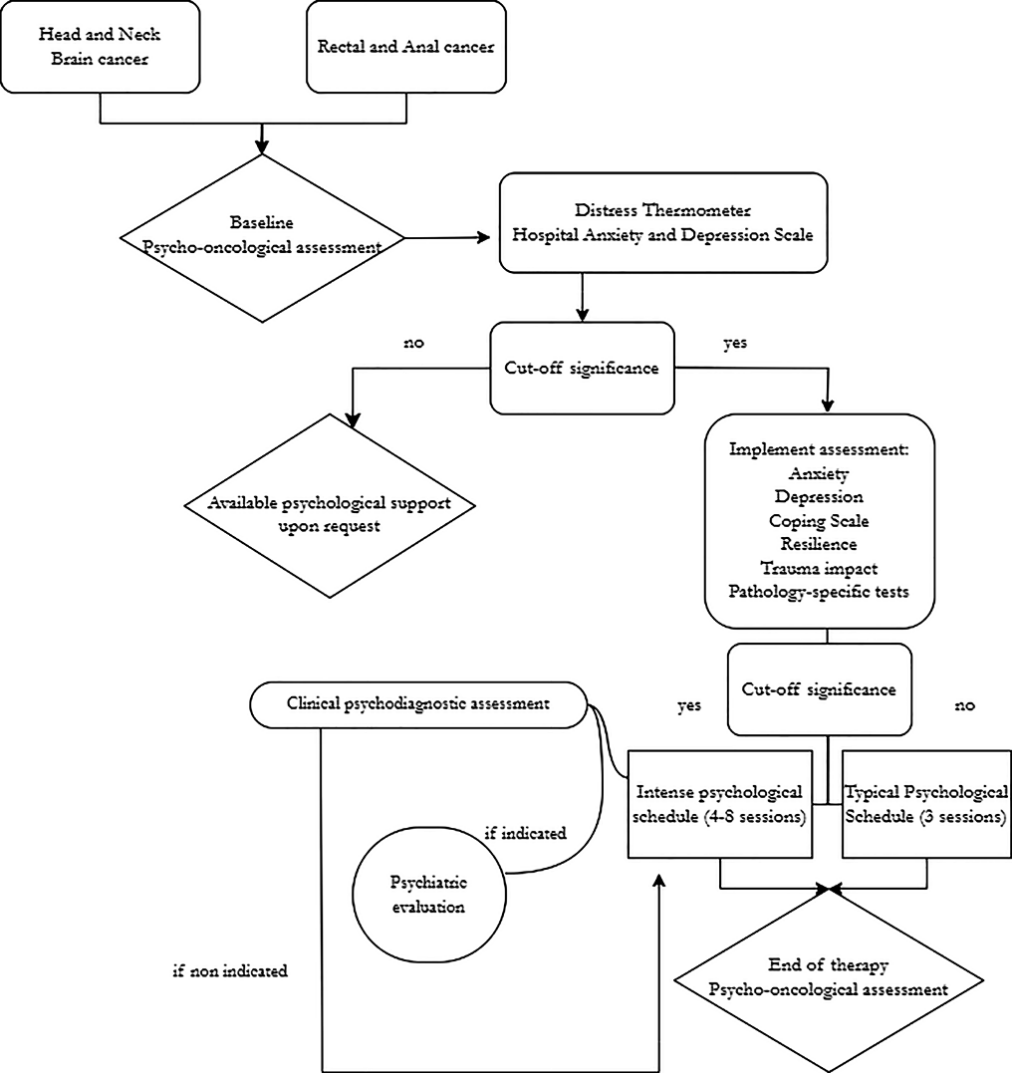
Flowchart representing personalized psycho-oncological care for head and neck, brain, rectal, and anal cancers patients in the art environment of Gemelli ART

The psychotherapeutic approach is based on the principles of Gestalt psychotherapy: it is i) relational and nonauthoritarian, ii) experiential and present centered, iii) humanistic and non-pathologizing, and iv) uses the sensation of the body as a foundation for connecting to moment-to-moment experiences [139]. In case of the traumatic impact of cancer on patients, psycho-traumatological paths are followed by means of eye movement desensitization and reprocessing psychotherapy [140, 141], a WHO-recommended approach to trauma [142]. The use of technology through apps or digital platforms can enable swift psychosocial support for patients undergoing RT. The flowchart in Fig. 4 represents the psycho-oncological support implemented at Gemelli ART. The support is aimed at all patients, with particular attention on head and neck, brain, rectal, and anal cancers.

### The Isola ART experience and the Terentium project

The Isola ART is the new center of excellence in oncology at the Ospedale Isola Tiberina—Gemelli Isola in Rome (also known as the Island of Medicine for its dedication to Asclepius, the ancient god of medicine, in 290 B.C). Guided by a scientific and clinical expertise often co-created with the Gemelli ART team, as well as human sensitivity thanks to its historical location and spiritual values, the Isola ART experience aims to convey humanistically guided medicine, where technology and arts support the healthcare team in providing the best clinical pathway to each cancer patient.

**Fig. 5:**
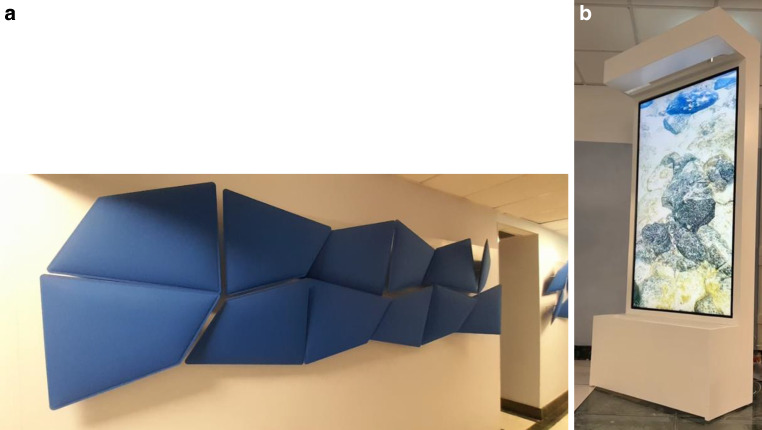
Digital renderings of the furniture that convey the metaphor of water. **a** The wall panels, designed with a metaphorical reference to water, were donated by Infinity Office Srl. **b** Donated by Senior l’Età della Saggezza ONLUS, the multisensorial fountain combines nature and technology. Patients can interact with it by choosing images that resonate with them, including some contributed by the patients

In the Radiation Oncology Unit, all patients receive the same welcoming and humanistic care from the healthcare team and administrative staff. The attention and care dedicated to them are complemented by artistic and digital furnishings which recall the metaphor of water (Fig. 5) as part of the standard of care. A gentle melody of natural sounds spreads within the waiting room and transforms the waiting time into a moment of relaxation and meditation.

It is worth mentioning that donations have been provided by workers who understand the importance of the Isola ART experience, as well as by patients who have been cured by our healthcare professionals and are willing to contribute with their creations/donations to the care of other patients, since they have perceived the role of art during their journey as relieving and valuable. This is a form of engagement where patients become ambassadors of care and testify that the humanistic care provided to them created a constructive meaning to their existential earthquake (see “Creation” section).

**Fig. 6:**
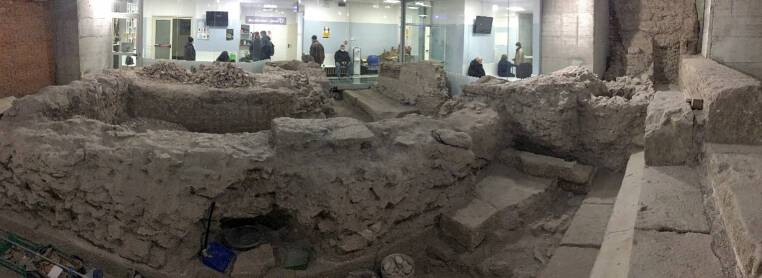
Inside view of the temple of Iuppiter Iurarius

Another element of wonder in patients is the presence of the temple of Iuppiter Iurarius (Jupiter the Oath-Taker) built in 194 B.C., an historical heritage that is carefully preserved and, at the same time, is a fascinating sight to those who visit the unit (Fig. 6). We are planning to put some interactive displays on the glasses (outside the temple) through which patients have the possibility to see a virtual reconstruction of the ancient temple.

**Fig. 7:**
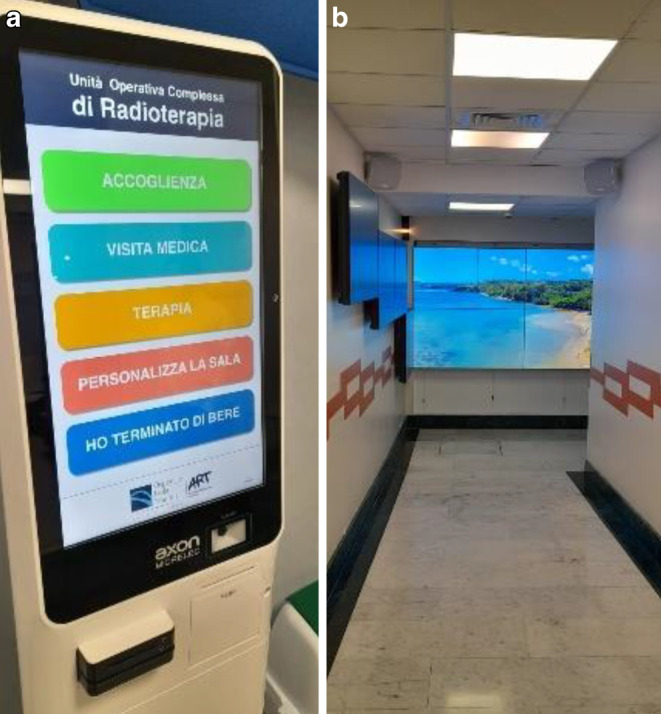
Personalized therapy room. **a** Patients can use the digital panel to choose a naturalistic/artistic video that they wish to see in the treatment room for the whole duration of RT. Translation of items selectable from the digital monitor: “Welcoming, Medical visit, Therapy, Personalize the room, I’ve finished drinking.” **b** Digital rendering of the immersive room with the wall monitors that display the video selected by the patient

Meanwhile, we have created an immersive room in which patients undergoing RT have the possibility to personalize the treatment room by selecting a video they wish to see during the whole therapy session in the room (Fig. 7).

In this context, we are planning to evaluate whether the context, from the welcome room to the treatment room, and the artistic stimuli can provide benefits to the patients (as suggested by the literature). Furthermore, we plan to conduct several studies to systematically assess—also using salivary cortisol levels—which kind of stimuli are most beneficial and to which kind of patient, in order to plan their reintegration into normal life by understanding how to increasingly personalize their pathway. For example, we could offer patients dedicated artistic stimuli at home through various technologies (e.g., apps, videos, music, paintings); this approach could also be used to collect PROMs and PREMs in order to predict and prevent any late toxicity or adverse event.

## Conclusion

Making sense of a disease is crucial, and art as well as technologies may help to facilitate this experience. However, these expedients themselves cannot convey empathy by design, since empathy is given when two human beings understand each other through the meaning of a shared common experience [143], nor be uplifting per se, because they require a meaningful relationship and a context in which the meaning is signified. As part of this framework, it is important to highlight the central role of gesture and human contact in soothing unpleasant emotions [144].

On the one hand, esthetic stimuli should be selected carefully since they may elicit different effects in different people [145]. In this scenario, art therapy is not conceived as going to a museum or a concert but rather as an expedient used within the clinical pathway as a dedicated tool for symptom relief and/or trauma elaboration, transforming the therapeutic care into an experience that enables the person to become empowered and encourages them to talk about the disease to their clinician/therapist when words are not enough.

On the other hand, information provided to patients for therapeutic/educational purposes should be always supervised and monitored; patients often refer to “Dr. Google” to understand their symptoms and diseases as a form of response to the existential earthquake they are experiencing and to make sense of their disorientation. However, disinformation caused by the infodemic leads patients to make detrimental choices for their health [146]. Nowadays, deepfake can emulate interviews of physicians on the TV and make them say harmful things about other therapies [147]. Trusting the news is becoming harder, and this is why building a trustful therapeutic relationship is essential. Still, technologies for health are not intended as “wellness apps” downloadable from online stores but rather as tools tested in research settings and integrated into the clinical pathway, while monitored by a team of experts who are in charge of the patient for therapeutic purposes [75].

Value should be attributed to the meaning of the disease for each specific patient, and personalization of the therapeutic pathway is always co-created with the patient towards gestures of care (smiling, active listening, empathy, compassion) that technologies essentially lack, since they do not experience human struggles like failure, heartbreaks, pain, loss [74, 99].

Art therapy and technologies can be ancillary supports for clinicians and not substitutive of their expertise and responsibilities. Such tools may help to convey a more empathetic and uplifting patient journey if properly integrated within clinical practice, where the humanistic touch of medicine remains pivotal. To this end, they are “allies, not enemies” when:

- their integration is proven to be trustworthy after passing strong clinical and scientific validation;
- their integration into clinical settings is explainable and the inclusion/exclusion criteria of such tools is transparent for the scientific community; and
- their integration is supported by consolidated evidence showing the real benefits for the care of oncology patients.

Clinicians and therapists remain those who choose whether to delegate their choices to technologies or not. Having a team integrated in the tumor board is fundamental for the overall assessment of the patient’s condition.
